# A gender comparison of psychological distress among medical students in Nigeria during the Coronavirus pandemic: a cross-sectional survey

**DOI:** 10.4314/ahs.v22i1.63

**Published:** 2022-03

**Authors:** Oluwaseun Mercy Idowu, OyinOluwa Gloria Adaramola, Boluwatife Samson Aderounmu, Ifeoluwa Delight Olugbamigbe, Olaoluwa Ezekiel Dada, Adeyinka Christopher Osifeso, Oluseun Peter Ogunnubi, Oluwakemi Ololade Odukoya

**Affiliations:** 1 College of Medicine, University of Lagos, Nigeria; 2 College of Medicine, University of Ibadan, Nigeria; 3 Lagos State University College of Medicine; 4 Department of Psychiatry, College of Medicine, University of Lagos, Nigeria; 5 Department of Community Health and Primary Care, College of Medicine, University of Lagos, Nigeria

**Keywords:** Psychological distress, psychological impact, gender, COVID-19, medical students

## Abstract

**Background:**

The Coronavirus disease (COVID-19) pandemic as a large scale stressor could have negative effects on the mental health of medical students. Since gender differences in mental health may exist, it is important to see if a large scale stressor like the pandemic may be associated with variances in the psychological distress between both genders.

**Objectives:**

To assess and compare the psychological distress of male and female medical students during the COVID-19 pandemic.

**Methods:**

A cross-sectional survey was carried out among 1010 medical students from three universities in southwestern Nigeria within the first six months of the first reported case of the COVID-19 pandemic. The respondents were purposively selected. Data was obtained online on participants' demographic and psychological distress using the General Health Questionnaire 12 (GHQ-12). Data was analyzed using the SPSS version 21, student t and chi-square tests were used to assess gender differences, and multivariate regression to assess the predictors of psychological distress among both genders. p values less than 0.05were considered statistically significant.

**Results:**

Overall, female participants (OR=1.455, 95% CI= 1.095–1.936) were twice more likely to have experienced psychological distress than males during the COVID-19 pandemic. Age (OR=0.922, 95% CI= 0.867–0.979), being in pre-clinical years (OR= 1.394, 95% CI= 1.004–1.938), having a family income less than 100,000 naira (OR= 1.379, 95% CI=1.442–6.723) a previous history of mental illness (OR=3.077, 95% CI= 1.430–6.615) and having a relative/acquaintance diagnosed with COVID 19(OR=1.646, 95% CI= 1.062–2.551) were independently associated with psychological distress among the respondents. When comparing both genders, among females, age (OR=0.886, 95% CI= 0.803–0.978), family income less than 100,000 naira (OR=1.898, 95% CI= 1.306–2.759) and a previous history of mental illness (OR=5.266, 95% CI= 1.894–14.635) were associated with psychological distress, while, being in pre-clinical years (OR= 1.713, 95% CI= 1.052–2.790) was associated with psychological distress among males.

**Conclusion:**

Females had more psychological distress compared to male students. It is recommended that gender-specific interventions addressing psychological distress among medical students are instituted.

## Introduction

Coronavirus disease 2019 (COVID-19) is caused by the severe acute respiratory syndrome coronavirus-2 (SARSCoV2), which is highly transmissible and has been a cause for worldwide concern.^1^ Since the outbreak of COVID-19 in Wuhan, China in December 2019,^2^ the virus has expressed high rates of rapid transmission.

It was declared a pandemic on the 11^th^ March, 2020 after more than 118,319 confirmed cases and 4,292 deaths had been recorded from various continents of the world.^3^ Shortly before this declaration, Nigeria, the most populous country in Africa, recorded her first case on the 27^th^ February, 2020.^4^ By the 24^th^ of October 2020, Nigeria had a total of 61,930 confirmed cases with 1,129 deaths. Almost half (49%) of these cases are in the southwest states of the country.^5^

According to the World Health Organization (WHO), mental health is “a state of well-being in which the individual realizes his or her own abilities, can cope with the normal stresses of life, can work productively and is able to make contributions to his or her community”.^6^ A person's mental health is influenced by social and economic conditions like family, school and social support among other factors.^7^ It is widely believed that social ties play a positive part in the preservation of one's mental health.^8^ In a bid to curb the spread of the COVID-19 there has been a disruption in social life due to the advent of measures like social distancing, lockdown, self-isolation and quarantine. Apart from social distancing, the possibility of fear and uncertainty in the hearts of many concerning the novel coronavirus may pose a risk to their mental health.^9^ More so, several nations have suffered from economic recessions as a result of the adverse distress of COVID-19 on the economy, leading to loss of employment or reduction in income which could negatively affect mental health.^10^

Gender differences in mental health may exist between males and females, with females observed to have a higher prevalence in mental health disorders compared to males.^11^ This may be because females are exposed to risk factors such as gender inequality, gender-based violence and gender discrimination.^11^ A mental health survey carried out in Lagos State, Nigeria revealed significant gender differences with females showing higher prevalence. 12 Since strategies for identification, prevention and treatment of mental disorders may be based on gender,^13^ highlighting the need for gender-specific studies when measuring the psychological distress of any population will be useful.

Previous studies have shown that the predisposing factors to mental health disorders may be gender specific.^14^–^17^ It is important to see if a large scale stressor such as the pandemic is associated with gender differences in th. This study therefore assessed and compared the psychological distress of COVID-19 among male and female medical students in medical schools in South-Western Nigeria. It also assessed the risk factors associated with psychological distress among male and female medical students. It is hoped that the study findings will be useful for policy makers in the educational sector and provide information useful in tailoring the medical curriculum to suit the peculiar needs of medical students during and after the COVID-19 pandemic.

## Materials and methods

This was a descriptive cross-sectional study that compared the psychological distress between male and female medical students of the three largest Colleges of Medicine in the South-western regions of Nigeria. The Colleges are: College of Medicine, University of Ibadan (COMUI), College of Medicine, University of Lagos (CMUL) and Lagos University Teaching Hospital (LASUCOM). Purposive sampling was used to select the three largest medical schools in South-western Nigeria, which the study was carried out. Convenience sampling method was used to recruit eligible male and female respondents (medical and dental undergraduates of the three universities), who participated in the study.

The minimum sample size suitable for this cross-sectional descriptive study was calculated using the formula for comparison between two groups:

$$\begin{array}{cc} \text{Sample size =} & \underline{\text{2SD2 (1.96+0}{\text{.84)}}^{\text{2}}}\\ & {\text{d}}^{\text{2}} \end{array}$$

SD = Standard deviation from previous studies (This was derived from ‘Gender Differences on Perceived Social Support and Psychological Distress among University Students’).^17^

d = effect size (difference between mean values)


$$\begin{array}{cc} \text{Sample size =} & \underline{\text{2(10)2(1.96+0}{\text{.84)}}^{\text{2}}}\\ & {\left(1.8\right)}^{\text{2}} \end{array}$$

Sample size =483.95

Hence, in this study we need 484 participants per group (male and female)

### Data collection

This survey was conducted from June 22 to July 16, 2020. This was about 4–5 months after the first case of COVID-19 were reported in Nigeria. Because it was not feasible to do a face-face sampling survey during the on-going pandemic, data was collected using an onlinesurvey platform (Google forms https://forms.gle/19y-fEzehJKwsme759). Relying on the authors' networks with colleagues in the three universities, a recruitment poster was created and posted to the class online platforms in the three colleges. This poster contained a brief introduction of the background, voluntary nature of participation, declarations of anonymity and confidentiality, as well as the link code of the online questionnaire. Persons, who are medical students of the three colleges and agreed to participate in the study were instructed to complete the questionnaire by clicking the link

### Survey instrument

The General Health Questionnaire (GHQ-12), a self-administered screening tool was used to assess individuals with psychological distress.^18^ It was developed in 1970 by Sir David Goldberg and Paul Williams;^19^ the twelve item GHQ-12 is the most extensively used screening instrument for common mental disorders, in addition to being a more general measure of psychiatric well-being. It assesses the severity of mental problems over the past few weeks. Its brevity makes it attractive for use.^18^,^19^ Its psychometric properties have been studied in various countries and in several sub-populations, including in Nigeria.^20^,^21^

### GHQ scoring

The customary types of scores used in the GHQ scoring system are a bimodal scale (0-0-1-1) and a 4-point Likerttype scale (0-1-2-3). The latter scale (0-0-1-1) produces a more acceptable distribution of scores for parametric analysis and was used to grade the participants' risk of psychological distress during the pandemic in our study. Using the 0-0-1-1scale, a score of 0 was awarded to ‘Not at all’ and ‘No more than usual’ responses while a score of 1 were awarded to ‘Same as usual’ and ‘much less than usual’ responses. Hence, a total score ranging from 0 to 12 was generated; in which positive items are corrected from 0 (always) to 1 (never) and the negative ones from 1 (always) to 0 (never), so that higher scores indicate worse mental health18We used a cut-off point of 3, which has been found reliable and is widely used locally in Southwest Nigeria.^19^ Scores ≤ 2 implied that the participants had ‘No risk of psychological distress’, whereas scores ≥ 3 implies that participants were ‘At risk of psychological distress’.

### Data analysis

Data collected was analyzed with SPSS 21 statistical software for windows (version 21.0 SPSS Inc, Chicago IL). Categorical variables were expressed in frequency tables with the corresponding percentages while normally distributed continuous variables were expressed as means and standard deviations. Chi-square was used to assess gender differences in categorical and continuous variables respectively. A multivariate regression analysis was used to identify the predictors of psychological distress first among the general participants, and subsequently for males and females separately. p values of <0.05 were considered statistically significant.

Ethical considerations: Ethical approval was obtained from Research and Ethics Committee of the Lagos University Teaching Hospital, with HREC assigned number: LUTHHREC/EREV/0620/56. Informed consent (online) was obtained from the participants before the commencement of the study. Participation was voluntary and Confidentiality assured to all respondents. Data was stored anonymously in a password-protected database.

## Results

The socio-demographic characteristics of study participants are shown in Table 1. The total number of data collected for male respondents was 486 and that of females, 524. The mean age of female respondents was 21.28 ± 2.5 years. More than half (54.7%) of the respondents with “family monthly income greater than 100,000 naira”, were females. Almost two-third (65.5%) of medical students “previously diagnosed with a mental condition” were also females. 64.7% of respondents who had “a relative/acquaintance diagnosed with COVID-19” were female medical students. In comparison, the mean age of male respondents was 22.43±3.2 years which is a little higher than the mean age of females. Male respondents constitute less than half (45.3%) of respondents with “family income greater than 100,000 naira”. Fewer males (34.5%) onstitute the group of respondents “diagnosed with a mental illness”, and 35.3% of respondents who had relatives/acquaintances diagnosed of COVID-19 were also males.

**Table 1:** Socio-demographics of Male and Female Participants

Variables	Male n=486	Female n=524	Total	Chi-square p value
	Frequency (%)	Frequency (%)		(X2)
Age group <25 >25 Mean ± SD =	465 (47.4) 21 (75.0) 22.43±3.221	517 (52.6) 7 (25.0) 21.28±2.523	982 (100) 28 (100)	X2= 35.386 p=< 0.001
Ethnicity Yoruba Igbo Hausa Edo [1]Others	362 (48.9) 82 (46.6) 3 (3.0) 12 (42.9) 27 (55.6)	379 (51.1) 94 (53.4) 0 (0.0) 16 (57.1) 35 (66.8)	741 (100) 176 (100) 3 (100) 28 (100) 62 (100)	X2= 36.645 p= 0.223
Religion Christianity Islam Others	400 (46.6) 80 (56.3) 6 (66.7)	459 (53.4) 62 (43.7) 3 (33.3)	859 (100) 142 (100) 9 (100)	X2= 5.913 p= 0.052
Institution COMUI CMUL LASUCOM	169 (56.5) 182 (44.4) 135 (43.9)	130 (43.5) 228 (55.6) 166 (55.1)	299 (100) 410 (100) 301 (100)	X2= 12.028 p= 0.002
Level Pre-clinical Clinical	199 (48.5) 287 (47.8)	211 (51.4) 313 (52.2)	410 (100) 600 (100)	X2= 4.636 p= 0.328
Marital Status Single Married	478 (48.3) 8 (64.3)	511 (51.7) 13 (35.7)	989 (100) 21 (100	X2= 0.958 p= 0.619
Family Monthly income <100,000 >100,000	258 (50.9) 228 (45.3)	249 (49.1) 275 (54.7)	507 (100) 503 (100)	X2= 3.425 p= 0.331
Have you ever been diagnosed with a mental illness? Yes No	10 (34.5) 476 (48.5)	19 (65.5) 505 (51.5)	29 (100) 981 (100)	X2= 2.224 p= 0.136
Do you have a Relative/Acquaintance with COVID-19 Yes No	36 (35.3) 450 (49.6)	66 (64.7) 458 (50.4)	102 (100) 908 (100)	X2= 7.475 p= 0.006

Abbreviations: SD Standard deviation, COMUI College of Medicine, University of Ibadan, CMUL College of Medicine, University of Lagos, LASUCOM Lagos State University Teaching Hospital.

### Psychological distress assessment

From the results in Table 2, female participants reported they had “lost much sleep over worry” (53.1%), “been feeling depressed” (56.2%), and “been thinking of themselves as worthless” (54.1%), ‘Rather more than usual’, which is higher than the reports from male participants (46.9%, 43.8%, and 45.9% respectively).

**Table 2:** Responses of the participants to GHQ-12 Questions. Have you during the COVID-19 pandemic:

Statement	Male n (%)	Female n (%)	Total	Chi-square p value
Been able to concentrate on what you're doing Better than usual (0) Same as usual (0) Less than usual (1) Much less than usual (1)	331 (48.4) 119 (48.1) 27 (45.0) 9 (45.0)	353 (51.6) 127 (51.6) 33 (55.0) 11 (55.0)	684 (100) 246 (100) 60 (100) 20 (100)	X2= 0.339 p= 0.593
Lost much sleep over worry Not at all (0) No more than usual (0) Rather more than usual (1) Much more than usual (1)	79 (49.4) 251 (48.3) 129 (46.9) 27 (49.1)	81 (50.6) 269 (51.7) 146 (53.1) 28 (50.9)	160 (100) 520 (100) 275 (100) 55 (100)	X2= 0.288 p= 0.962
Felt that you're playing a useful part in things More than usual (0) Same as usual (0) Less than usual (1) Much less than usual (1)	90 (49.2) 239 (48.2) 117 (46.2) 40 (49.4)	93 (50.8) 257 (51.8) 133 (53.8) 41 (50.6)	183 (100) 496 (100) 250 (100) 81 (100)	X2= 0.309 p= 0.958
Felt capable of making decisions about things More than usual (0) Same as usual (0) Less than usual (1) Much less than usual (1)	114 (47.1) 320 (47.8) 38 (48.1) 14 (70.0)	128 (52.9) 349 (52.2) 41 (51.9) 6 (30.0)	242 (100) 669 (100) 79 (100) 20 (100)	X2=3.957 p=0.266
Felt constantly under strain Not at all (0) No more than usual (0) Rather more than usual (1) Much more than usual (0)	188 (48.2) 194 (47.4) 78 (49.7) 26 (48.1)	202 (51.8) 215 (52.6) 79 (50.3) 28 (51.9)	390 (100) 409 (100) 157 (100) 54 (100)	X2= 0.232 p= 0.972
Felt you could not overcome your difficulties Not at all (0) No more than usual (0) Rather more than usual (1) Much more than usual (1)	301 (48.1) 136 (47.9) 36 (51.4) 13 (43.3)	325 (51.9) 148 (52.1) 34 (48.6) 17 (56.7)	626 (100) 284 (100) 70 (100) 13 (100)	X2= 0.589 p= 0.899
Been able to enjoy your normal day to day activities More than usual (0) Same as usual (0) Less than usual (1) Much less than usual (1)	85 (47.0) 243 (47.6) 135 (48.3) 23 (48.2)	96 (53.0) 268 (52.4) 126 (51.7) 33 (41.8)	181 (100) 511 (100) 261 (100) 56 (100)	X2=2.634 p= 0.451
Been able to face up your problems More than usual (0) Same as usual (0) Less than usual (1) Much less than usual (1)	103 (47.5) 315 (49.1) 57 (44.9) 11 (44.0)	114 (52.5) 326 (50.9) 70 (55.1) 14 (56.0)	217 (100) 641 (100) 127 (100) 25 (100)	X2= 1.009 p= 0.799
Been feeling unhappy or depressed Not at all (0) No more than usual (0) Rather more than usual (1) Much more than usual (1)	250 (47.6) 149 (48.9) 57 (43.8) 30 (40.0)	275 (52.4) 156 (51.1) 73 (56.2) 20 (60.0)	525 (100) 305 (100) 130 (100) 50 (100)	X2= 3.896 p= 0.273
Been losing confidence in yourself Not at all (0) No more than usual (0) Rather more than usual (1) Much more than usual (1)	298 (47.8) 121 (49.6) 49 9 (47.6) 18 (46.2)	325 (52.2) 123 (50.4) 54 (52.4) 21 (53.8)	623 (100) 244 (100) 103 (100) 39 (100)	X2= 0.304 p= 0.959
Been thinking of yourself as a worthless person Not at all (0) No more than usual (0) Rather more than usual (1) Much more than usual (1)	383 (48.4) 64 9 (47.1) 28 (45.9) 11 (48.1)	408 (51.6) 72 (52.9) 33 (54.1) 11 (51.9)	791 (100) 136 (100) 61 (100) 22 (100)	X2= 0.241 p= 0.971
Been feeling reasonably happy all things considered More than usual (0) Same as usual (0) Less than usual (1) Much less than usual (1)	105 (49.3) 291 (47.0) 71 (49.7) 19 (54.3)	108 (50.7) 328 (53.0) 72 (50.3) 16 (45.7)	213 (100) 619 (100) 143 (100) 35 (100)	X2= 1.090 p= 0.780

### Psychological distress

Results from Table 3 showed that 60.3% of medical students “At risk” of psychological distress were females, which is higher compared to male students (39.7%).

**Table 3:** Respondents showing risk of Psychological distress

Psychological distress	Male n (%)	Female n (%)	Total	Chi- square p value
No risk	360 (51.8)	333 (48.1)	693 (100)	**X^2^= 12.969** **p= <0.001**
At risk	126 (39.7)	191 (60.3)	317 (100)	
Total	486 (48.1)	524 (51.9)	1010 (100)	

### Predictors of psychological distress among both genders

From Table 4; Age (OR=0.922, p=0.009, 95% CI= 0.867–0.979), female gender (OR=1.455, p=0.010, 95% CI= 1.095–1.936), pre-clinical levels (OR= 1.394, p= 0.048, 95% CI= 1.004–1.938), family income less than #100,000 (OR= 1.379, p= 0.026, 95% CI=1.442–6.723) positive history of mental illness (OR=3.077, p=0.004, 95% CI= 1.430–6.615) and relative/acquaintance diagnosed with COVID 19(OR=1.646, p= 0.026, 95% CI= 1.062–2.551) were independently associated with psychological distress among all respondents in general. The institution of the respondents was not significantly associated with psychological distress of participants generally.

**Table 4:** Socio-demographics and predictors of psychological distress among all respondents

Variables	Adjusted OR	Std. err.	z score	p value	95% CI
Constant	1.467	1.072	0.52	0.600	0.350–6.141
Age in years	0.922	0.029	-2.61	0.009	0.867–0.979
Female gender	1.455	0.211	2.58	0.010	1.095–1.936
Level of Study Pre-clinical	1.394	0.234	1.98	0.048	1.004–1.938
Family monthly income <100,000 naira	1.379	0.194	2.28	0.026	1.442–6.723
Positive history of mental illness	3.077	1.202	2.88	0.004	1.430–6.615
Institution CMUL (Ref) LASUCOM COMUI	1.148 0.855	0.191 0.148	0.83 -0.90	0.409 0.367	0.828–1.592 0.609–1.201
Relative/Acquaintance diagnosed with COVID 19	1.646	0.368	2.23	0.026	1.062–2.551

Abbreviations: OR odds ratio, Std. err. Standard error, CI confidence interval. Constant estimates baseline odds

### Predictors of psychological distress among female respondents only

Table 5 shows that age (OR=0.886, p=0.017, 95% CI= 0.803–0.978), family income less than 100,000 (OR=1.898, p=0.001, 95% CI= 1.306–2.759) and positive history of mental illness (OR=5.266, p=0.001, 95% CI= 1.894–14.635) were independently associated with psychological distress among female participants. The institution of the respondents was not significantly associated with psychological distress of females.

**Table 5:** Socio-demographics and predictors of psychological distress among female respondents

Variables	Adjusted OR	Std. err.	z score	p value	95% CI
Constant	4.209	4.727	1.28	0.201	0.466–38.031
Age in years	0.886	0.045	-2.40	0.017	0.803–0.978
Level of Study Pre-clinical	1.178	0.278	0.69	0.488	0.741–1.872
Family monthly income <100,000 naira	1.898	0.362	3.36	0.001	1.306–2.759
Positive history of mental illness	5.266	2.746	3.19	0.001	1.894–14.635
Institution CMUL (Ref) LASUCOM COMUI	1.161 1.014	0.255 0.244	0.68 0.06	0.497 0.952	0.755–1.786 0.634–1.624
Relative/Acquaintance diagnosed with COVID 19	1.572	0.443	1.61	0.108	0.466–38.031

Abbreviations: OR odds ratio, Std. err. Standard error, CI confidence interval. Constant estimates baseline odds

### Predictors of psychological distress among male respondents only

From Table 6, only pre-clinical level (OR= 1.713, p= 0.031, 95% CI= 1.052–2.790) was independently associated with psychological effects among male participants. The institution of the respondents was not significantly associated with psychological distress of male respondents. Female respondents had higher risk of psychological distress compared to males. However, the predictors of psychological distress among the female participants are age, family income lessthan 100,000 naira and a positive history of mental health condition. Whereas, there was no predictor of psychological distress among male respondents.

**Table 6:** Socio-demographics and predictors of psychological distress among male respondents

Variables	Adjusted OR	Std. err.	z score	p value	95% CI
Constant	0.733	0.684	-0.33	0.739	0.117–4.562
Age in years	0.959	0.038	-1.04	0.300	0.887–1.037
Level of Study Pre-clinical	1.713	0.426	2.16	0.031	1.052–2.790
Family monthly income <100,000 naira	0.929	0.199	-0.34	0.733	0.610–1.416
Positive history of mental illness	1.441	1.023	0.51	0.609	0.355–5.841
Institution CMUL (Ref) LASUCOM COMUI	1.131 0.708	0.293 1.182	0.47 -1.34	0.636 0.179	0.679–1.881 0.428–1.172
Relative/Acquaintance diagnosed with COVID 19	2.004	0.746	1.87	0.062	0.966–4.159

Abbreviations: OR odds ratio, Std. err. Standard error, CI confidence interval. Constant estimates baseline odds

## Discussion

Our study revealed generally that, female participants, respondents in pre-clinical levels, with a positive history of mental illness, having relatives/acquaintances diagnosed with COVID-19 and family income less than #100,000 were independently associated with psychological distress. In comparison to the male participants, socio-demographic factors associated with psychological distress among females were age, family income less than #100,000 and positive history of mental illness, whereas, pre-clinical level was associated with the male participants

The findings in our study showed that the pandemic lead to psychological distress among the respondents. This result finding agrees with Gupta et al, which conducted a survey among people in Nepal, which reported that the participants had higher level of stress, anxiety and depression on the participants during the pandemic lockdown.^22^ The psychological distress displayed by our respondents was evidenced by loss of sleep, being depressed and loosing self-confidence. While our study agrees with Gupta et al, it however, contradicts the claims of Yun Li et al,^23^ where all participants had low scores for anxiety and depression. This unusual finding may be because, the numbers of COVID-19 cases at the onset of the pandemic were fewer compared to the latter period.

Our study also reports that there is a significant gender difference in the psychological distress of medical students with females constituting a majority (60%) of the population with high scores for psychological distress. This finding agrees with existing evidence from Ochilbek et al, conducted among African university students, which reports that females were at a higher risk of psychological distress than males.^24^ The study among African university students revealed a significant gender difference, as women had higher anxiety and depression scores than men which confirms our results.^24^ Furthermore, findings from our study supports a study conducted among the general population in Spain, which also reported that women showed significantly higher levels in anxiety, stress and depression compared to men.^25^ Additionally, a similar survey among health workers in Wuhan, found out that female healthcare workers were more exposed to the psychological threat of COVID-19 than male healthcare workers.^26^ However, the significant gender difference in this study is in contrast with findings from a similar pre-pandemic study which showed gender not to be significant in the psychological impact on the students. This may be because their study was done in the absence of a large scale stressor such as the pandemic.^27^

Our results from the regression analysis indicated that female participants with family income less than #100,000 ($200) are at risk of psychological distress compared to female participants who had higher family income. This is consistent with a study done among the United States community which discovered that income level was uniquely inversely associated with anxiety, financial worry and loneliness during the COVID-19 pandemic.^28^ Our study also agrees with a study among children and adolescent in China, which found out that the psychological problems of children and adolescents was mainly associated with loss of father's job, financial losses of family and unavailability of basic life needs.^29^ Furthermore, our regression analysis showed that female respondents who have been previously diagnosed with a mental disorder are also at increased risk of psychological distress, compared to female respondents who have never been diagnosed of a mental health condition. This finding further buttresses the theory that there is an association between mental disorders and stressful events.

## Limitations

This study is among the first few gender comparative studies in Nigeria to assess the psychological distress of the COVID-19 pandemic on medical students, it also features a large sample size of 1010 Nigerian medical students. However, it has some limitations and the findings should be interpreted with some caution. Information obtained was collected using an online self-administered questionnaire indicating the possibility of selection bias. Furthermore, the results cannot be generalized to the entire country because it was limited to participants in three universities only in the South West region of Nigeria. There is also a possibility for recall bias and causal inferences cannot be made since due to the cross-sectional nature of the study. A more representative sample and a broader study involving colleges of medicine across the entire country would provide more generalized and accurate estimate of the gender comparison of the psychological distress of COVID-19 in medical students.

## Conclusion

Females were more at risk of psychological distress compared to male students emphasizing the need for gender-specific interventions for psychological distress among medical students. Also, future research to establish the long term psychological effects of the COVID-19 pandemic on medical students is recommended.

The protected time for the contribution of OOO towards the research reported in this publication was supported by the Fogarty International Center of the National Institutes of Health under the Award Number K43TW010704. The content is solely the responsibility of the authors and does not necessarily represent the official views of the National Institutes of Health.
